# Cooperative phenotype predicts climate change belief and pro-environmental behaviour

**DOI:** 10.1038/s41598-022-16937-2

**Published:** 2022-07-26

**Authors:** Scott Claessens, Daniel Kelly, Chris G. Sibley, Ananish Chaudhuri, Quentin D. Atkinson

**Affiliations:** 1 School of Psychology, University of Auckland, Auckland, New Zealand; 2 Department of Economics, University of Auckland, Auckland, New Zealand; 3 CESifo, Munich, Germany

**Keywords:** Psychology, Human behaviour

## Abstract

Understanding the psychological causes of variation in climate change belief and pro-environmental behaviour remains an urgent challenge for the social sciences. The “cooperative phenotype” is a stable psychological preference for cooperating in social dilemmas that involve a tension between individual and collective interest. Since climate change poses a social dilemma on a global scale, this issue may evoke similar psychological processes as smaller social dilemmas. Here, we investigate the relationships between the cooperative phenotype and climate change belief and behaviour with a representative sample of New Zealanders (*N* = 897). By linking behaviour in a suite of economic games to self-reported climate attitudes, we show robust positive associations between the cooperative phenotype and both climate change belief and pro-environmental behaviour. Furthermore, our structural equation models support a motivated reasoning account in which the relationship between the cooperative phenotype and pro-environmental behaviour is mediated by climate change belief. These findings suggest that common psychological mechanisms underlie cooperation in both micro-scale social dilemmas and larger-scale social dilemmas like climate change.

## Introduction

Climate change belief varies considerably across individuals, both within and between countries^1,2^. While the majority of people in developed countries accept the reality of anthropogenic climate change, considerable minorities are either undecided, accept that the climate is changing but deny a human role, or deny that it is changing at all^3–5^. Individual differences also exist in the uptake of pro-environmental behaviour such as energy conservation^6,7^ and environmental activism^8^. Understanding the underlying psychological causes of this variation will help us determine whether and how increased numbers can be encouraged to act.

One psychological mechanism that could explain variation in climate change belief and pro-environmental behaviour is a general willingness to cooperate in social dilemmas. Social dilemmas are classes of social interaction in which an actor’s self-interest is at odds with the group’s collective interest^9^. A classic example is the commons dilemma^10^, often associated with Hardin’s “tragedy of the commons”^11^. When a resource is collectively-held, individuals must choose between maximising their own benefit (i.e., defecting) or restraining themselves to sustain the resource for everyone (i.e., cooperating). Maximising individual benefit delivers short-term profits, but eventually leads to the collapse of the resource.

Studies using incentivised behavioural economic games have revealed a general psychological preference for cooperation in micro-scale social dilemmas that is temporally stable^12,13^, heritable^14,15^, and captured by a single underlying latent variable that is found across a variety of cultures^13,16,17^. Dubbed the “cooperative phenotype”, this measure of an individual’s willingness to cooperate and behave prosocially in micro-scale social dilemmas correlates with self-reported moral values, positive views regarding real-world cooperation (i.e., paying taxes), and manifest helping behaviour^13^.

Given what we know about the preferences, beliefs, and behaviours of individuals with cooperative phenotypes in smaller social dilemmas, it is conceivable that much of this knowledge can be applied to climate change beliefs and pro-environmental behaviour. This is because climate change shares the structure of a social dilemma, albeit at a much larger scale. Self-interested behaviour erodes the shared commons of a stable climate, delivering individually beneficial results that are eventually ruinous for all^18^. In contrast, tackling climate change requires extensive cooperation on a global scale. Parties must take on personal costs in order to support the public good of a stable climate, and ensure that such behaviour is shared by sufficient numbers to achieve its aim^19,20^.

Evidence suggests that common psychological mechanisms are used to navigate both micro-scale and larger scale social dilemmas. For example, one study^21^ conducted two-player public goods games in forest commons user groups and found that groups with a greater share of conditional cooperators (defined as those whose extent of cooperation is positively correlated with their beliefs about the cooperativeness of their peers) in the games had a significantly higher percentage of crop trees per hectare. In other words, cooperators in the economic games were more successful at cooperating to manage large forest commons.

Here, we consider an analogous question concerning the much larger, more complex social dilemma of climate change. We predict that individual differences in the cooperative phenotype will explain variation in both pro-environmental behaviour and belief in climate change. First, those unwilling to engage in costly cooperation, especially where interactions are short-lived and future benefits small or non-existent, will be less willing to behave pro-environmentally, as doing so involves paying personal costs to benefit the collective. Second, we predict that non-cooperators will also be less likely to believe in the reality of climate change: cognitively, it is easier to justify uncooperative behaviour by refusing to admit that there is a social dilemma at all. This is an example of motivated reasoning^22–24^, a form of reasoning in which people process information in a biased manner to form beliefs that reinforce their existing predispositions (in this case, their cooperative predispositions). Motivated reasoning has been highlighted as a key factor in the formation of climate change beliefs^25^.

In recent decades, research has begun to test the relationships between cooperative behaviour, pro-environmental behaviour, and climate change belief. Several studies have found that self-reported social value orientation predicts pro-environmental behaviour^26–29^. However, these studies did not report associations with climate change belief. They also used self-reported measures of prosociality, which may be subject to social desirability bias and therefore might not accurately reflect people’s behavioural cooperative phenotype. In experimental economics, incentivised economic games are preferred to self-report measures when eliciting private behavioural preferences^30,31^. Research with economic games has found that cooperative behaviour across different micro-scale social dilemmas is positively related to self-reported pro-environmental behaviour^26,29,32,33^ (but see Ref.^34^ who found no relationship between cooperative decisions and pro-environmental behaviour). By contrast, only one study that we know of has estimated the association between gameplay and climate change belief^20^. In that case, all games were explicitly framed to participants as “climate dilemmas”, preventing any investigation of an association between climate change belief and the micro-scale social dilemma structure of the games alone.

In this pre-registered study (https://osf.io/d8t46/), we combined data on self-reported climate change belief and pro-environmental behaviour from a longitudinal study of attitudes and values with an expanded suite of the economic games used to estimate individuals’ cooperative phenotypes. Given the structural similarity between micro-scale social dilemmas and environmental problems, we first hypothesised that the cooperative phenotype would predict pro-environmental behaviour. Second, in line with our argument for motivated reasoning, we also hypothesised that the cooperative phenotype would predict climate change belief, and that pro-environmental behaviour would mediate this relationship. In testing these hypotheses, we aimed to establish whether the relationships between the cooperative phenotype and climate change belief and behaviour are independent of factors previously shown to relate to climate attitudes, such as gender, age, education, and political affiliation^35–37^ and personality^38–41^.

Participants were sampled from the New Zealand Attitudes and Values Study, a nationally representative survey of registered voters in New Zealand containing socio-demographic data, personality scales, and measures of self-reported pro-environmental behaviour and climate change belief. Participants were screened for eligibility before playing a suite of incentivised one-shot economic games online with other participants in real-time (*N* = 897). We used four economic games commonly utilised in behavioural economics to model different micro-scale social dilemmas. Three games, previously used to validate and estimate the cooperative phenotype^13^, measured cooperative and prosocial behaviour (Dictator Game, Trust Game, Public Goods Game). A fourth game not used in previous research^13^ measured coordination with others (Stag Hunt Game). The cooperative phenotype was estimated by fitting confirmatory factor analyses to the data from all four of these economic games, before running a series of structural equation models testing our main hypotheses.

## Methods

### Power analysis

In order to determine a minimum size for our sample, we conducted a power analysis using existing data from a previous study^13^, setting our effect size from the smallest significant correlation between economic game play and real-world cooperation (*r* = 0.15). To detect this correlation effect size with statistical power of 0.95, the power analysis software G*Power^42^ suggested a sample size of 571 participants. We aimed to sample 1000 participants, considerably more than suggested, to deal with potential dropouts alongside a longitudinal study.

### Participants and sampling

Participants were sampled from the ongoing New Zealand Attitudes and Values Study, a nationally-representative longitudinal study drawn from the New Zealand electoral roll. We included participants in our sample frame who: had completed Wave 9 and/or Wave 10 (*n* = 8095); had not subsequently withdrawn from the New Zealand Attitudes and Values Study at the time of sampling (*n* = 7833); had indicated that they were willing to take part in further online studies (*n* = 4181); had a valid email (*n* = 4040); lived in New Zealand (*n* = 3955); were younger than 70 at the time of sampling (*n* = 3374); and had a valid phone contact (*n* = 3345). Out of this total of 3345 participants, 2731 were successfully contacted about a further study on “economic decision-making in groups”.

Following contact, participants who agreed to take part were sent follow-up emails to arrange a time to take part in a battery of online economic games. 1686 participants either dropped out of the study at this stage (were uninterested, unavailable, or ceased replying) or were excluded for failing to complete the games. In order to focus on the largest population at a single time slice, we only retained participants from Wave 9 (*n* = 1045). Finally, participants were excluded for taking too little (less than 5 min) or too much (more than 50 min) time to complete the games (*n* = 2), or for failing to answer the relevant items on climate change belief and pro-environmental behaviour (*n* = 147). This left us with a final sample of 897 participants (612 females; age M = 51 years, SD = 12 years).

### Materials

#### New Zealand Attitudes and Values Study measures

Main dependent variables and covariates were taken from Wave 9 of the New Zealand Attitudes and Values Study, because this wave measured the environmental attitudes of interest. Climate change belief was assessed with three items^5^: “Climate change is real”; “Climate change is caused by humans”; and “I am deeply concerned about climate change”. Items were rated on a 7-point Likert scale, from 1 (strongly disagree) to 7 (strongly agree). Pro-environmental behaviour was assessed using a single item^43^, rated on the same 7-point Likert scale: “Have you made sacrifices to your standard of living (e.g., accepted higher prices, driven less, conserved energy) in order to protect the environment?”.

In addition, we used data on a number of key socio-demographic variables (age, gender, ethnicity, education level, and political party support). Political party support was assessed on 7-point Likert scales for each major New Zealand party^44^. These were then converted into a single categorical variable, reflecting the party with the highest support. Education was assessed on a 10-point ordinal rank scale in accordance with the New Zealand Qualifications Framework^45^. We also used mean scores for self-report items measuring seven key personality dimensions: extraversion, agreeableness, conscientiousness, neuroticism, openness to experience, honesty-humility, and narcissism. Self-report personality items were taken from the Mini-IPIP6^46^ and rated on 7-point Likert scale. See Supplementary Materials for full self-report items from the New Zealand Attitudes and Values Study.

#### Economic games

Eight economic games were conducted using oTree software^47^. These were selected to replicate existing research and are largely identical to those used in a previous study^13^. The games all involve one-shot decisions between multiple players for points corresponding to real world stakes (1 point = NZD $0.035), with the strategy method used to induce responses across all possible roles. Game code and a copy of the text for the games can be found online at https://osf.io/d8t46/. While the full study also contained games that measure norm-enforcing punishment, in this study we focus on the four games that measure cooperation and coordination.

Three games measure cooperation and prosociality, in which participants must choose between individual pay-off and taking on a personal cost in order to benefit others.

- *Dictator game.* Player A receives 100 points and must decide how many (if any) to transfer to Player B, who is passive. Any points not transferred are kept by Player A.
- *Trust game.* Players A and B both receive 50 points. Player A starts and, with the understanding that the transferred amount will be tripled, is given the choice to transfer all 50 points to Player B. If Player A transfers their 50 points, Player B receives 150 points, taking their total to 200. Player B then has the option to transfer 0–150 points back to Player A.
- *Public goods game.* Four players receive 100 points each, and are given the option to contribute 0–100 points into a common pool. Players decide at the same time, then the amount in the common pool is doubled and shared evenly amongst all four players. Each player finishes with the amount they retained after the decision to contribute, as well as their share from the common pool.

The final relevant game focuses on coordination, and replaces the destructive All-Pay Auction Game used in previous work^13^ in order to see if the cooperative phenotype extends to coordination behaviour.

- *Stag hunt game.* Four players each receive 50 points. Players choose between contributing 30 points into a shared group project or contributing nothing. Decisions are made simultaneously. All points in the group project are doubled and distributed evenly amongst the players, but only if all players contributed. Failing this, all points in the group project are lost. Each player finishes with their share from the group project, plus the points they retained following their contribution.

### Procedure

Data collection for economic game responses took place weekly between the 18th of February 2019 and the 25th of July 2019, utilising a staggered recruitment model. Following expressions of interest in an initial phone call, participants were emailed further information and asked to complete a Qualtrics survey. This allowed participants to specify their availability for testing in a specific session the following week, while excluding respondents who lacked adequate Internet access, a quiet place to participate in the study, or a New Zealand bank account for payment purposes.

Game sessions took place on midweek evenings from 6 to 8 pm, and varied in size between 14 and 97 participants. At the specified time of testing, participants received an email containing a link to oTree. Once on the website, participants entered their unique code before filling out a consent form informing them of ethical approval, their confidentiality and right to withdraw, and how they would be reimbursed. Following agreement, participants then read information about the economic games, including the real-world stakes and real-time matching with other participants.

The eight games (cooperation and norm-enforcing punishment games) were then presented in a random order, with participants reading specific instructions and answering comprehension questions for each game in turn before providing responses for all possible roles in the game. Once the games had been completed, participants entered a waiting lobby until all other participants were finished. The software then calculated payoffs for each game by randomly matching participants in each session. Players were shown a summary screen with payoffs for each game as well as their total accumulated payoff.

In situations where sessions did not contain multiples of four (due to drop-out or availability), simulated players were used to make up the shortfall with their responses based on median responses from previous work^13^. Participants were informed of this possibility at the end of the session, after all game decisions had been elicited: “In the rare event that we could not find a participant to match you with, we have instead matched you with average decisions based on previous research”.

Each participant’s final payoff consisted of the accumulated payoffs from all eight games (between NZD $10 and $35; *M* = $25.20, *SD* = $2.45), plus a fixed $20 show-up fee. Name and bank account details were collected at the end of the study, encrypted and stored online before being decrypted on a local computer for payment.

Participants took an average of 22 min to complete the eight games (SD = 7 min, range = 6–47 min). There was a 50 min threshold for game completion. Due to the demands of real-time matching between participants, those who took longer than 50 min were progressed to the waiting lobby, and treated as if they were simulated players. Participants who timed out still received the $20 show-up fee, but no bonus payment.

### Statistical analyses

Our pre-registered analyses consisted of confirmatory factor analyses and structural equation modelling (https://osf.io/d8t46/). We fitted confirmatory factor analyses (CFAs) to both the economic game data and our measures for climate change belief. We estimated the “cooperative phenotype” as a latent variable with factor loadings from the Dictator Game, Trust Game (Give), Trust Game (Return), Public Goods Game, and Stag Hunt Game. We estimated “climate change belief” as a latent variable with factor loadings from three items: “Climate change is real”; “Climate change is caused by humans”; and “I am deeply concerned about climate change”.

We then fitted a series of structural equation models testing our main hypotheses. First, we regressed pro-environmental behaviour on the “cooperative phenotype”. Second, we regressed “climate change belief” on the “cooperative phenotype”. Third, we ran a mediation analysis testing whether pro-environmental behaviour mediated the relationship between “cooperative phenotype” and “climate change belief”, and subsequently reversed this mediation in an exploratory analysis. For all hypotheses, we controlled for socio-demographic and personality variables that have previously been shown to be related to environmental attitudes, namely age, gender, ethnicity, political party support, education, extraversion, agreeableness, conscientiousness, neuroticism, openness to experience, narcissism, and honesty/humility^35–41^.

We pre-registered that we would exclude participants who failed *any* of the comprehension questions for the four cooperation games. This resulted in a large reduction in sample size (from 897 to 574) and a potentially biased sample. To maximise statistical power, we opted to retain those who had failed the comprehension questions and control for comprehension directly in our structural equation models. However, our main results were unchanged when following the pre-registered exclusion criteria (see Supplementary Results). We additionally pre-registered that we would re-run our main analyses with cooperation in the Public Goods Game as the predictor variable. Results were largely unchanged in these analyses, although we found evidence for full mediation in both structural equation mediation models (see Supplementary Results).

All analyses were conducted in R Version 4.0.2^48^. The *lavaan* package^49^ was used for fitting confirmatory factor analyses and structural equation models, the *ggplot2* package^50^ was used for visualisation, and the *drake*^51^ and *papaja*^52^ packages were used to reproducibly generate the manuscript.

### Ethics statement

Ethical approval for this study was granted by the University of Auckland Human Participants Ethics Committee (ref: 021666). The study was performed in accordance with all the relevant guidelines and regulations. Informed consent was obtained from all participants prior to the study.

## Results

**Figure 1:**
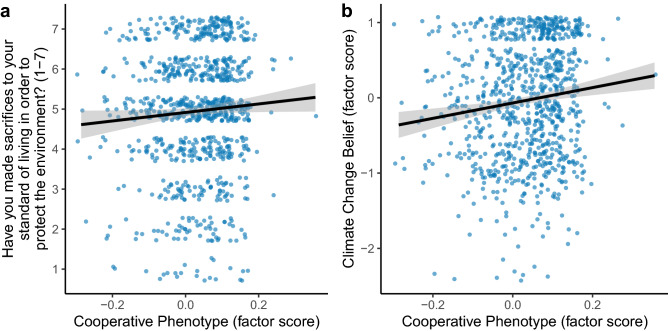
Cooperative phenotype positively predicts both pro-environmental behaviour (**a**) and belief in climate change (**b**). “Cooperative phenotype” is a latent variable captured by cooperative decisions in the Dictator Game, Public Goods Game, Trust Game, and Stag Hunt Game. “Climate change belief” is a latent variable captured by three self-report items measuring belief in the reality of climate change, belief that climate change is human caused, and concern about climate change. For visualisation ease, regression lines and 95% confidence interval shaded areas are predictions from least-squares regressions without covariates.

In line with our pre-registered hypotheses, we found a significant positive relationship between the cooperative phenotype and self-reported pro-environmental behaviour (unstandardised *b* = 0.75, 95% CI [0.09 1.40], *r* = 0.10, *p* = 0.025; Fig. 1a). Individuals who cooperated more in our economic games modelling micro-scale social dilemmas were more likely to report engaging in pro-environmental behaviour than individuals who cooperated less. We also found a positive relationship between the cooperative phenotype and climate change belief (*b* = 1.08, 95% CI [0.43 1.74], *r* = 0.16, *p* = 0.001; Fig. 1b). Individuals who cooperated more were more likely to believe in anthropogenic climate change than individuals who cooperated less. This positive relationship held when separately analysing the individual items making up the climate change belief latent variable in exploratory models: belief in the reality of climate change (*b* = 1.04, 95% CI [0.29 1.78], *r* = 0.14, *p* = 0.006), belief that climate change is human-caused (*b* = 1.03, 95% CI [0.33 1.73], *r* = 0.14, *p* = 0.004), and concern about climate change (*b* = 1.09, 95% CI [0.38 1.80], *r* = 0.14, *p* = 0.003).

**Figure 2:**
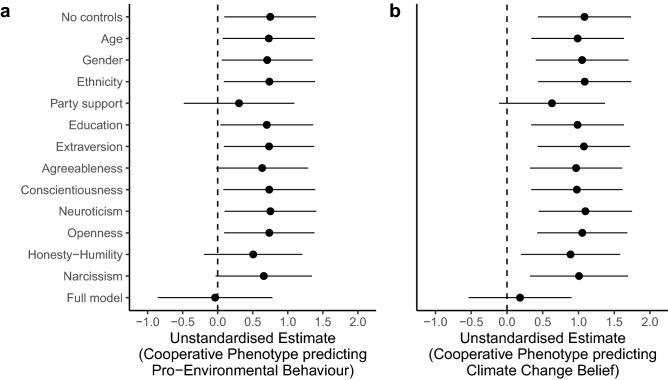
Controlling for socio-demographic and personality variables. (**a**) The unstandardized estimate for the relationship between the cooperative phenotype factor and pro-environmental behaviour, across various models controlling for different socio-demographic and personality variables. (**b**) The unstandardized estimate for the relationship between the cooperative phenotype factor and climate change belief. Lines represent 95% confidence intervals.

In order to investigate the relationship between these effects and other potential causal factors, we re-ran our models controlling for socio-demographic and personality variables previously shown to predict climate change belief. Regarding socio-demographic variables, the relationship between the cooperative phenotype and self-reported pro-environmental behaviour was robust to controls for age, gender, ethnicity, and education, but was attenuated by political party supported (Fig. 2a). We found the same attenuating effect of political party for climate change belief (Fig. 2b). Regarding personality variables, the relationship between the cooperative phenotype and self-reported pro-environmental behaviour was robust to controls for extraversion, conscientiousness, neuroticism, and openness, but was attenuated by agreeableness, honesty-humility, and narcissism. In contrast, the relationship between the cooperative phenotype and climate change belief was robust to the inclusion of all personality covariates, suggesting that this result is independent of previously identified personality effects^38–41^.

**Figure 3:**
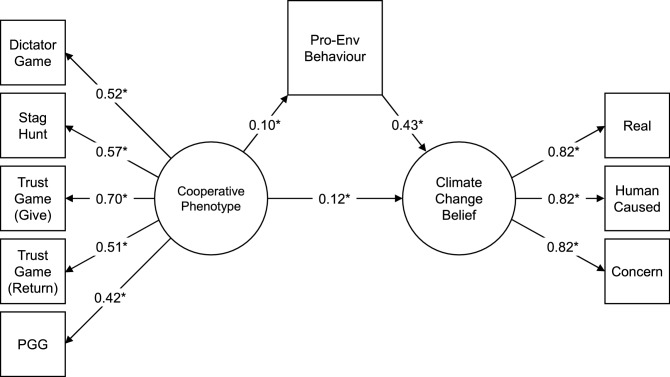
Structural equation mediation model (N = 897). Regressing the climate change belief factor on the cooperative phenotype factor, mediated by pro-environmental behaviour. *Note:* this visualisation does not include paths from the full model predicting game behaviour from game comprehension. Numbers are standardised parameter estimates; *p < 0.05.

To test for an effect of motivated reasoning, whereby the cooperative phenotype affects pro-environmental behaviour and thus one’s willingness to believe in the reality of climate change, we fitted a mediation model investigating whether pro-environmental behaviour mediated the relationship between cooperative phenotype and climate change belief. This model fitted the data well (RMSEA = 0.038; SRMR = 0.052; CFI = 0.987; Fig. 3). Regressing pro-environmental behaviour on the cooperative phenotype was statistically significant (*b* = 0.76, 95% CI [0.10 1.43], standardised *β* = 0.10, *p* = 0.025), as was regressing climate change belief on pro-environmental behaviour (*b* = 0.37, 95% CI [0.32 0.42], *β* = 0.43, *p* < 0.001). However, while including pro-environmental behaviour as a mediator did decrease the unstandardised parameter for the direct path between cooperative phenotype and climate change belief, this relationship remained significant (*b* = 0.77, 95% CI [0.19 1.36], *β* = 0.12, *p* = 0.010). Some, but not all, of the relationship between cooperative phenotype and climate change belief can be explained by pro-environmental behaviour as a mediator. This pattern of results held when controlling for all socio-demographic and personality covariates except agreeableness, honesty-humility, and narcissism, which attenuated the path from the cooperative phenotype to pro-environmental behaviour, and political party support, which attenuated both paths from the cooperative phenotype to climate change belief and behaviour.

**Figure 4:**
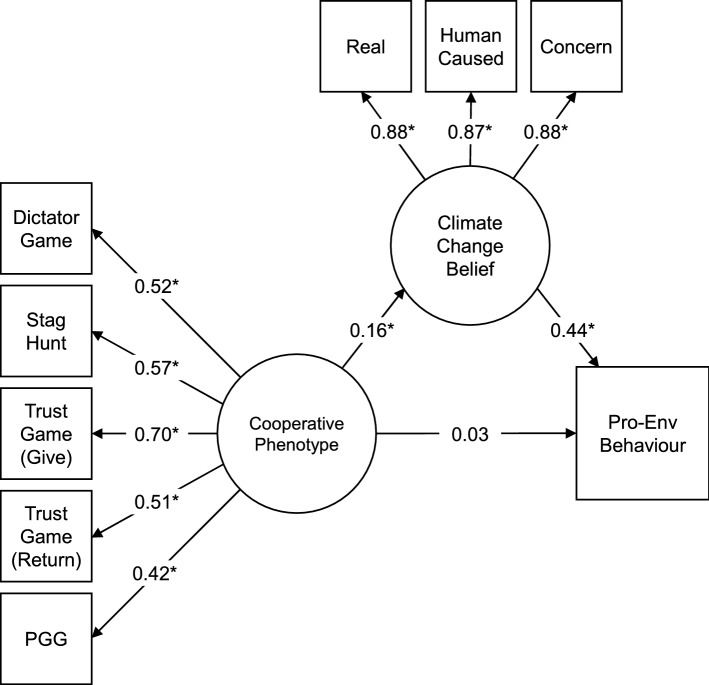
Reversed structural equation mediation model (N = 897). Regressing pro-environmental behaviour on the cooperative phenotype factor, mediated by the climate change belief factor. *Note:* this visualisation does not include paths from the full model predicting game behaviour from game comprehension. Numbers are standardised parameter estimates; *p < 0.05.

An alternative motivated reasoning account could be that people directly update their belief in climate change based on their cooperative preferences, which in turn causes pro-environmental behaviour. To explore this, we swapped the climate change belief and behaviour variables in an exploratory reversed mediation model. This reversed mediation model fitted the data slightly better than the initial model (*∆* SRMR = − 0.014; Fig. 4). In this model, regressing climate change belief on the cooperative phenotype was significant (*b* = 1.06, 95% CI [0.42 1.70], *β* = 0.16, *p* = 0.001) as was regressing pro-environmental behaviour on climate change belief (*b* = 0.50, 95% CI [0.44 0.57], *β* = 0.44, *p* < 0.001). Moreover, including climate change belief as a mediator removed the significance of the direct path between the cooperative phenotype and pro-environmental behaviour (*b* = 0.23, 95% CI [− 0.39 0.86], *β* = 0.03, *p* = 0.467), showing that the total effect of the cooperative phenotype on pro-environmental behaviour is mediated by climate change belief. These results therefore provide greater support for an alternative motivated reasoning model in which the cooperative phenotype directly predicts belief in climate change, which in turn encourages pro-environmental behaviour^53^. This pattern of results held when controlling for all socio-demographic and personality covariates except political party support, which attenuated the path from the cooperative phenotype to climate change belief.

The attenuating effect of political party support throughout all of our main analyses suggests that the cooperative phenotype and political party support share common variance. In a final exploratory analysis, we regressed the cooperative phenotype onto political party support. In particular, we analysed reported support for the major political parties in New Zealand: the progressive Green Party, the centre-left Labour Party, the centre-right National Party, and the socially conservative New Zealand First Party. We found that, relative to Green Party supporters, significantly lower cooperative phenotype scores were found for supporters of both National (*b* = − 0.09, 95% CI [− 0.13 − 0.05], *p* < 0.001) and Labour (*b* = − 0.05, 95% CI [− 0.09 − 0.01], *p* = 0.007) parties. This suggests that the broad prosocial tendency tapped by the cooperative phenotype may also explain some of the variance in political party support, which is itself an important predictor of climate change belief and pro-environmental behaviour.

## Discussion

These results demonstrate that the cooperative phenotype has positive, significant relationships with both pro-environmental behaviour and climate change belief. The more an individual cooperates in micro-scale social dilemmas, the more likely they are to both report cooperating in the large-scale dilemma of climate change and to believe in its reality. In contrast to claims that a positive link between economic gameplay and climate change belief was simply the result of the game’s explicit framing^20^, our results suggest that this previously observed correlation was due in part to more general similarities between the game’s payoff structure and that of the large-scale social dilemma of climate change. In addition, these results bolster support for the external validity of anonymous one-shot economic games as measures of real-world cooperation, a link which has been previously questioned^54^.

Despite this, the effect sizes linking the cooperative phenotype to climate change belief and pro-environmental behaviour were small. This likely reflects the complexity of these variables and the numerous interacting factors that produce them^55^. A tendency to cooperate in anonymous one-shot social dilemmas is only one aspect of how people form beliefs and act in the real world. Moreover, effect sizes for relationships between behavioural tasks and self-report measures tend to be small^56^. Nevertheless, the explanatory power of the cooperative phenotype on climate change belief and behaviour is comparable to other socio-demographics deemed important in previous work, such as age, gender, and ethnicity^35,37^ (Supplementary Fig. 1).

In contrast to our hypothesised motivated reasoning account of climate change belief, whereby behavioural preferences influence behaviour which in turn influences beliefs, we found more support for an alternative motivated reasoning model whereby the cooperative phenotype predicted climate change belief directly, which in turn predicted pro-environmental behaviour. Of course, our cross-sectional design does not allow us to make causal claims with these data. Nevertheless, this model is more in line with the theory of planned behaviour^57^, which posits that normative beliefs produce behavioural intentions. This fits with other findings suggesting that climate change beliefs are important motivators of pro-environmental behaviour change^58,59^.

In our models, the variable that explained the largest proportion of variance in both climate change belief and behaviour was political party support (Supplementary Fig. 1). This corroborates research highlighting that political affiliation can be a strong predictor of climate change belief^35^, even in New Zealand, where climate change is not as politicised as places like the United States. The relationships between the cooperative phenotype and our dependent variables were also consistently attenuated by the inclusion of political party support (Fig. 2). This was because New Zealand political parties differed significantly in the cooperative phenotypes of their supporters: we found supporters of the progressive environmentally-focussed Green Party had significantly higher cooperative phenotype scores than supporters of both the centre-right National Party and the centre-left Labour Party. More work is needed to understand why individuals with different social preferences are drawn to different ends of the political spectrum^60^. Despite only small differences between political parties, these between-group differences can potentially have a dramatic effect when it comes to the formation of policy. For example, while centre-right National supporters may only slightly favour motorway construction over investment in rail, and vice versa for centre-left Labour, these small between-group differences can become magnified during the process of in-group deliberation^61^ leading to group opinions more extreme than those held by any individual members. Similarly, slightly higher cooperative phenotype levels in the Green Party as opposed to National or Labour may provide the between-group differences necessary for group polarisation to produce divergent policy on climate change.

Our findings show that how people in a developed Western democracy feel about climate change and whether or not they engage in pro-environmental behaviour is predicted by a general cooperative preference that is expressed in even abstract micro-scale social dilemmas. This same preference also appears to shape or be shaped by political party support, though the causal relationships between these variables remain unclear. Future work should seek to clarify the directions of causality, perhaps by exploiting longitudinal study designs that identify causation through changes over time. Such work should use comprehensive self-report scales and observations of real world pro-environmental behaviour^62^ to expand our findings beyond three-item and single-item measures of climate change belief and behaviour. Research should also evaluate the generalisability of these findings by extending our work to other cultures and by including further measures of cooperative and moral preferences (e.g. preferences for “doing the right thing”^63^). Regardless, if we are correct that the same psychological mechanisms underlie cooperation in both micro-scale and large-scale social dilemmas, then many of the behavioural nudges shown to promote cooperation in micro-scale social dilemmas^64^, such as reputation^65,66^, social norms^67^, sanctioning^68,69^, and stable localised interactions^70^, also have the potential to encourage people to believe in and act on climate change. Dedicated policy-based research programs^71^ and meta-analytic studies^72^ will be required to determine whether these factors could also be applied to promote cooperation in the large-scale social dilemma of climate change.

## Supplementary Information

Supplementary Information.

## Data Availability

A copy of the anonymous data reported in each New Zealand Attitudes and Values Study publication is available from Professor Chris Sibley (c.sibley@auckland.ac.nz) upon request from appropriately qualified researchers. Such data will be provided with the explicit understanding that it is used solely for the purposes of replicating or otherwise checking the validity of analyses reported in scientific papers analysing New Zealand Attitudes and Values Study data.

## Code availability

Python code for the incentivised behavioural tasks and R code for the statistical analyses, figures, and manuscript generation are publicly available at https://osf.io/d8t46/.
