# Supplementary material for: Cooperative phenotype predicts climate change belief and pro-environmental behaviour
Source: Sci Rep. 2022 Jul 26;12:12730. doi: 10.1038/s41598-022-16937-2 (PMC9325867; doi:10.1038/s41598-022-16937-2)
Supplement: Supplementary file 1 — Supplementary Information. [file 41598_2022_16937_MOESM1_ESM.pdf]

Supplementary Materials

Cooperative phenotype predicts climate change belief and pro-environmental behaviour

Scott Claessens[1], Daniel Kelly[1], Chris G. Sibley[1], Ananish Chaudhuri[2,3], & Quentin D. Atkinson[1]

[1] School of Psychology, University of Auckland, Auckland, New Zealand

[2] Department of Economics, University of Auckland, Auckland, New Zealand

[3] CESifo, Munich, Germany

Correspondence concerning this article should be addressed to Quentin D. Atkinson, Floor 2, Building 302, 23 Symonds Street, Auckland, 1010, New Zealand. E-mail: q.atkinson@auckland.ac.nz

Supplementary Results

Confirmatory factor analyses (CFAs). Before testing our hypotheses, we fitted two CFA models. In order to assess the "cooperative phenotype" (Peysakhovich et al., 2014), we fitted a confirmatory factor analysis model that loaded participant responses across our five game outcomes (Cronbach's α = 0.54): the Dictator Game, Trust Game (Give), Trust Game (Return), the Public Goods Game, and the Stag-Hunt Game. Instead of removing participants who failed the games' respective comprehension questions by listwise deletion, we took advantage of the structural equation modelling approach and controlled for comprehension by including each different game's comprehension question in the model. We then investigated model fit using two popular absolute measures of fit. The Root Mean Square Error of Approximation (RMSEA) was 0.05, indicating a good model fit (MacCallum, Browne, & Sugawara, 1996), and the Standardized Root Mean Square Residual (SRMR) was 0.05, also indicating a good model fit (Hu & Bentler, 1999). This step was important to validate our further analyses.

We then fitted a CFA model that loaded participant responses to our three different measures of climate change belief (Cronbach's α = 0.85): whether climate change is real, whether it is caused by humans, and the degree to which it is a concern. The model was just-identified, and therefore was perfectly fit to the data (RMSEA = 0.00; SRMR = 0.00).

Proportion of variance explained. Analyses of the variation explained by our models (R^2) reveal that cooperative phenotype alone accounts for 1.02% of the variation in pro-environmental behaviour and 2.58% of the variation in climate change belief. Similar patterns hold for both of these models: while the variation explained by cooperative phenotype is small, it is comparable to that explained by other variables in our sample that have been shown to be significant predictors of climate change belief, such as age, gender, and ethnicity (Hornsey et al.,

2016; Supplementary Figure 1). For example, in our sample, age accounts for 3.35% of the variation in climate change beliefs, while gender accounts for 0.62% and ethnicity accounts for 0.29% of the variation in comparison to 2.58% for the cooperative phenotype. However, not only does political party support attenuate the effect of cooperative phenotype on both climate change belief and pro-environmental behaviour, it also accounts for a far larger proportion of the variance in these variables: 25.62% for climate change belief, and 7.70% for pro-environmental behaviour.

Main analyses with exclusions. We pre-registered that we would exclude participants who failed *any* of the comprehension questions for the four cooperation games. This resulted in a reduced sample of 574 participants.

When including only these participants, we found a significant positive relationship between the cooperative phenotype and self-reported pro-environmental behaviour (unstandardised b = 0.84, 95% CI [0.08 1.61], r = 0.12, p = .029), and a significant positive relationship between the cooperative phenotype and climate change belief (unstandardised b = 1.13, 95% CI [0.36 1.90], r = 0.18, p = .004).

We then tested a mediation model with pro-environmental behaviour mediating the relationship between the cooperative phenotype and climate change belief (as in Figure 3). We found a significant direct effect of the cooperative phenotype on climate change belief (b = 0.78, 95% CI [0.08 1.47], standardised β = 0.12, p = .029). We also found that the cooperative phenotype predicted pro-environmental behaviour (b = 0.84, 95% CI [0.09 1.60], standardised β = 0.12, p = .029), and pro-environmental behaviour predicted climate change belief (b = 0.37, 95% CI [0.30 0.43], standardised β = 0.41, p < .001).

Finally, we fitted an exploratory reversed mediation model, where climate change belief mediated the relationship between the cooperative phenotype and pro-environmental behaviour (as in Figure 4). We found no direct effect of the cooperative phenotype on pro-environmental behaviour once we included climate change belief as a mediator (b = 0.33, 95% CI [-0.39 1.05], standardised β = 0.05, p = .372). However, the cooperative phenotype predicted climate change belief (b = 1.09, 95% CI [0.35 1.83], standardised β = 0.12, p = .004), and climate change belief predicted pro-environmental behaviour (b = 0.48, 95% CI [0.40 0.55], standardised β = 0.43, p < .001).

Main analyses with Public Goods Game. We also pre-registered that we would run versions of our models that additionally replaced the cooperative phenotype with cooperative behaviour in the Public Goods Game (PGG; i.e., amount contributed to the shared project). We report these results here.

We found a significant positive relationship between PGG cooperation and self-reported pro-environmental behaviour (unstandardised b = 0.36, 95% CI [0.08 0.63], r = 0.11, p = .011), and a significant positive relationship between PGG cooperation and climate change belief (unstandardised b = 0.28, 95% CI [0.01 0.55], r = 0.10, p = .042).

We then tested a mediation model with pro-environmental behaviour mediating the relationship between PGG cooperation and climate change belief. We found no direct effect of PGG cooperation on climate change belief (b = 0.14, 95% CI [-0.12 0.39], standardised β = 0.05, p = .286). However, we found that PGG cooperation predicted pro-environmental behaviour (b = 0.36, 95% CI [0.08 0.63], standardised β = 0.11, p = .011), and pro-environmental behaviour predicted climate change belief (b = 0.38, 95% CI [0.32 0.45], standardised β = 0.43, p < .001).

Finally, we fitted an exploratory reversed mediation model, where climate change belief mediated the relationship between PGG cooperation and pro-environmental behaviour. We found no direct effect of PGG cooperation on pro-environmental behaviour (b = 0.22, 95% CI [-0.04 0.49], standardised β = 0.07, p = .097). However, PGG cooperation predicted climate change belief (b = 0.28, 95% CI [0.01 0.54], standardised β = 0.05, p = .042), and climate change belief predicted pro-environmental behaviour (b = 0.48, 95% CI [0.40 0.56], standardised β = 0.43, p < .001).

Supplementary Figures

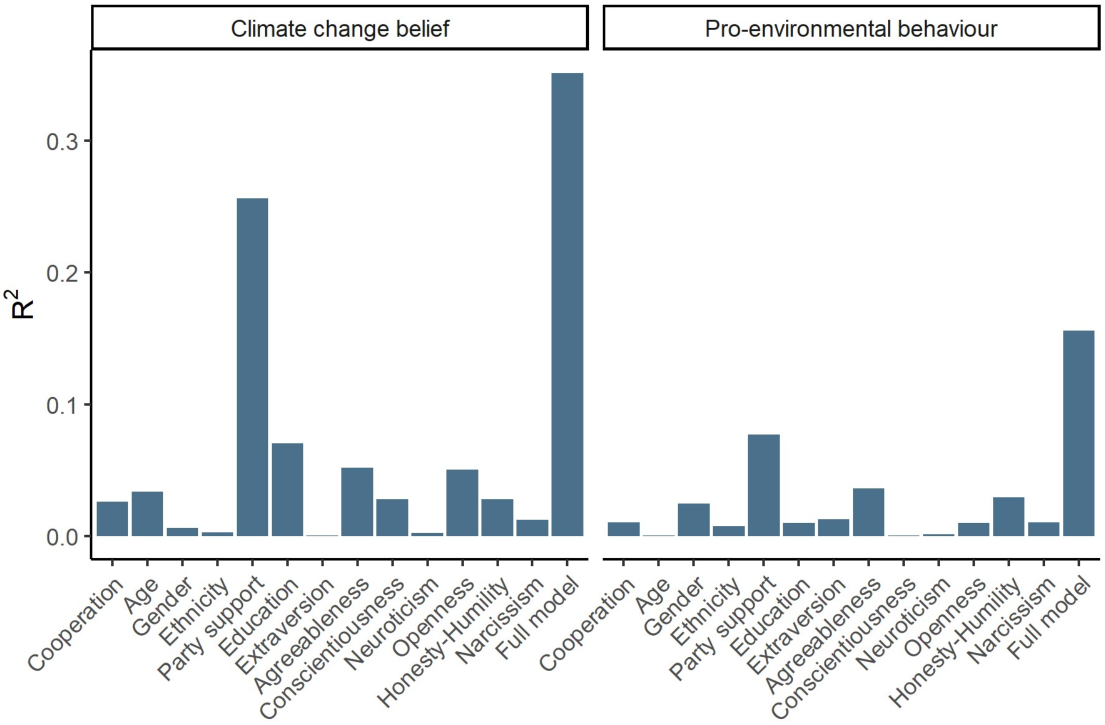

Supplementary Figure 1. Histograms comparing the proportion of variance in our dependent variables explained (R^2) by the cooperative phenotype, various socio-demographic and personality controls individually, and their combination in the full model.

Supplementary Tables

Supplementary Table 1. Self-report items from the New Zealand Attitudes and Values Study.

**Item**	**Description / Text**
Climate change belief	Climate change is real
	Climate change is caused by humans
	I am deeply concerned about climate change
Pro-environmental behaviour	Have you made sacrifices to your standard of living (e.g., accepted higher prices, driven less, conserved energy) in order to protect the environment?
Age	What is your date of birth?
Gender	What is your gender? (open-ended)
Ethnicity	Which ethnic group do you belong to? (NZ census question)
Education level	NZ Reg (0-10 education ordinal rank)
Political party support	Please rate how strongly you oppose or support each of the following political parties… the National Party
	Please rate how strongly you oppose or support each of the following political parties… the Labour Party
	Please rate how strongly you oppose or support each of the following political parties… the Green Party
	Please rate how strongly you oppose or support each of the following political parties… the NZ First Party
Extraversion	Am the life of the party
	Don't talk a lot (reversed)
	Keep in the background (reversed)
	Talk to a lot of different people at parties
Agreeableness	Sympathize with others' feelings
	Am not interested in other people's problems (reversed)
	Feel others' emotions
	Am not really interested in others (reversed)
Conscientiousness	Get chores done right away
	Like order
	Make a mess of things (reversed)
	Often forget to put things back in their proper place (reversed)
Neuroticism	Have frequent mood swings
	Am relaxed most of the time (reversed)
	Get upset easily
	Seldom feel blue (reversed)
Openness to experience	Have a vivid imagination
	Have difficulty understanding abstract ideas (reversed)
	Do not have a good imagination (reversed)
	Am not interested in abstract ideas (reversed)
Narcissism	Feel entitled to more of everything
	Deserve more things in life
Honesty/Humility	Would like to be seen driving around in a very expensive car (reversed)
	Would get a lot of pleasure from owning expensive luxury goods (reversed)

Supplementary References

Hornsey, M. J., Harris, E. A., Bain, P. G., & Fielding, K. S. (2016). Meta-analyses of the determinants and outcomes of belief in climate change. *Nature Climate Change*, 6(6), 622–626. https://doi.org/10.1038/nclimate2943

Hu, L., & Bentler, P. M. (1999). Cutoff criteria for fit indexes in covariance structure analysis: Conventional criteria versus new alternatives. *Structural Equation Modeling*, 6(1), 1—55. https://doi.org/10.1080/10705519909540118

MacCallum, R. C., Browne, M. W., & Sugawara, H. M. (1996). Power analysis and determination of sample size for covariance structure modeling. *Psychological Methods*, 1(2), 130—149. https://doi.org/10.1037/1082-989x.1.2.130

Peysakhovich, A., Nowak, M. A., & Rand, D. G. (2014). Humans display a 'cooperative phenotype' that is domain general and temporally stable. *Nature Communications*, 5(1), 1—8. https://doi.org/10.1038/ncomms5939
